# Baicalein, 7,8-Dihydroxyflavone and Myricetin as Potent Inhibitors of Human Ornithine Decarboxylase

**DOI:** 10.3390/nu12123867

**Published:** 2020-12-17

**Authors:** Yun-Chin Liu, Yi-Liang Liu, Ju-Yi Hsieh, Chang-Hsu Wang, Chi-Li Lin, Guang-Yaw Liu, Hui-Chih Hung

**Affiliations:** 1 Department of Life Sciences, National Chung Hsing University, Taichung 402, Taiwan; poooorme@yahoo.com.tw (Y.-C.L.); sgl0220@gmail.com (Y.-L.L.); crab882000@gmail.com (J.-Y.H.); johnny055279@gmail.com (C.-H.W.); 2 Institute of Medicine, College of Medicine, Chung Shan Medical University, Taichung 402, Taiwan; dll@csmu.edu.tw; 3 Division of Allergy, Immunology, and Rheumatology, Chung Shan Medical University Hospital, Taichung 402, Taiwan; 4 Institute of Genomics and Bioinformatics, National Chung Hsing University, Taichung 402, Taiwan; 5 iEGG and Animal Biotechnology Center, National Chung Hsing University, Taichung 402, Taiwan

**Keywords:** ornithine decarboxylase, chemoprevention, molecular docking simulations: baicalein, 7,8-dihydroxyflavone, myricetin

## Abstract

Background: Human ornithine decarboxylase (ODC) is a well-known oncogene, and the discovery of ODC enzyme inhibitors is a beneficial strategy for cancer therapy and prevention. Methods: We examined the inhibitory effects of a variety of flavone and flavonol derivatives on ODC enzymatic activity, and performed in silico molecular docking of baicalein, 7,8-dihydroxyflavone and myricetin to the whole dimer of human ODC to investigate the possible binding site of these compounds on ODC. We also examined the cytotoxic effects of these compounds with cell-based studies. Results: Baicalein, 7,8-dihydroxyflavone and myricetin exhibited significant ODC suppression activity with IC_50_ values of 0.88 µM, 2.54 µM, and 7.3 µM, respectively, which were much lower than that of the active-site irreversible inhibitor α-DL-difluoromethylornithine (IC_50_, the half maximal inhibitory concentration, of approximately 100 µM). Kinetic studies and molecular docking simulations suggested that baicalein, and 7,8-dihydroxyflavone act as noncompetitive inhibitors that are hydrogen-bonded to the region near the active site pocket in the dimer interface of the enzyme. Baicalein and myricetin suppress cell growth and induce cellular apoptosis, and both of these compounds suppress the ODC-evoked anti-apoptosis of cells. Conclusions: Therefore, we suggest that the flavone or flavonol derivatives baicalein, 7,8-dihydroxyflavone, and myricetin are potent chemopreventive and chemotherapeutic agents that target ODC.

## 1. Introduction

Ornithine decarboxylase (ODC; EC 4.1.1.17) is the first and rate-limiting enzyme in the biosynthesis of polyamines (putrescine, spermidine, and spermine). The biological functions of ODC and polyamines, such as embryonic development, cell differentiation, cell growth, cancer proliferation, cell transformation and aging, have been extensively studied [1,2,3,4,5,6]. The cellular levels of ODC are tightly regulated by a variety of physiological events [7].

Cellular levels of ODC and polyamines are frequently dysregulated in cancer and are associated with a critical role in cell proliferation [8,9,10,11,12]. In addition, ODC is able to reduce the accumulation of intracellular reactive oxygen species (ROS), protecting cancer cells against cellular apoptosis and therefore maintaining cancer cell survival [4,5]. ODC is considered an oncogene because its enzymatic activity leads to cancer initiation and proliferation [2,13]. Targeting oncogenic ODC could manipulate cell cycle checkpoints and protect against tumorigenesis. Therefore, ODC is a potential cancer target, and the discovery of ODC enzyme inhibitors is a beneficial strategy for cancer therapy and prevention [14,15]. An active-site irreversible inhibitor of ODC, α-DL-difluoromethylornithine (DFMO; also called eflornithine), has been approved by the FDA as a therapeutic agent for cancer treatment. DFMO has also been successfully used against other diseases, such as African sleeping sickness and a number of tropical diseases [16]. Additionally, it is widely used against hirsutism in women [17].

ODC is a short-lived protein that is rapidly degraded by the 26S proteasome within 30 min. ODC activity and degradation are delicately controlled by antizyme (AZ), which is a natural ornithine decarboxylase inhibitor that binds, inhibits, and promotes the protein degradation of ODC by the 26S proteasome [18,19]. High polyamine concentrations trigger a +1 ribosomal frameshifting of AZ mRNA to generate mature full-length AZ proteins [20,21,22]. In addition, c-Myc is the gene upstream of ODC, suggesting a strong association between the oncogene myc and the overexpression of ODC [23].

ODC is functional as a homodimer with its two active sites at the dimer interface that includes residues from each subunit [24]. ODC is inactivated by AZ binding, causing dissociation of the ODC dimer into monomers to form an inactive AZ-ODC heterodimer, finally promoting ODC degradation through a ubiquitin-independent proteasomal pathway [1,20,25]. Another regulatory protein of ODC, antizyme inhibitor (AZI), has a similar structure to that of ODC but lacks decarboxylase activity [26,27]. AZI binds with a greater affinity to AZ than ODC, thus rescuing ODC activity by liberating the ODC monomer from the inactive ODC-AZ heterodimer to reform active ODC dimers [25,28,29].

Although DFMO is an approved FDA drug against ODC, high doses of DFMO lead to hearing loss [30]. Therefore, discovering a new ornithine decarboxylase inhibitor with fewer side-effects would be quite valuable for chemoprevention or cancer therapy.

The discovery of agents from natural products that prevent cancer is a vital issue. A number of flavonoids have been investigated for their anticancer activities in a variety of cancer cell lines and animal models. The flavonol herbacetin has been found to be an allosteric inhibitor of ODC with anticancer activity [31]. By targeting ODC, herbacetin exerts activity against colon cancer and melanoma [31,32]. Baicalein (5,6,7-trihydroxyflavone) is a flavone that was originally isolated from the roots of Scutellaria baicalensis and Scutellaria lateriflora. 7,8-Dihydroxyflavone (7,8-DHF) is another natural flavone found in Godmania aesculifolia, Tridax procumbens, and primula tree leaves. Myricetin is easy to obtain from dietary sources, including vegetables, nuts, berries, oranges, grapes, and tea. Baicalein, 7,8-DHF and myricetin demonstrate diverse biological activities, including inhibiting cell proliferation, angiogenesis, and tumor metastasis; attenuating the neoplastic transformation of cancer cells; inducing cell cycle arrest, autophagy and apoptosis; targeting the mitochondria; and regulating enzymatic activity [33,34,35]. Additionally, these flavonoids protect against several human diseases, such as Alzheimer’s, Parkinson’s, cardiovascular pathologies, bone-related disorders, eye disorders and aging, and also have analgesic and antihypertensive activity [36,37,38].

In the present study, we provide evidence suggesting that baicalein, 7,8-DHF, and myricetin are potent inhibitors of human ODC, either in in vitro or in cell-based studies. This article also reveals the structure-activity relationships between ODC and these flavonoids through molecular docking simulations. Baicalein, 7,8-DHF, and myricetin are thus convincing candidates for the development of chemopreventive or chemotherapeutic agents.

## 2. Materials and Methods

### 2.1. Materials

Flavone, 7,8-DHF, 6,7-DHF, chrysin, pinocembrin, 6-hydroxyflavone, 7-hydroxyflavone, 7,8-dimethoxyflavone, flavonol, luteolin, and kaempferol were purchased from ChromaDex (Irvine, CA, USA). Baicalein, myricetin, quercetin, morin, galangin, apigenin, biochanin, rutin, naringenin, pyridoxamine, 4-pyridoxic acid, and 4-deoxypyridoxine were purchased from Sigma-Aldrich (St. Louis, MO, USA).

### 2.2. Cell Culture

The human promyelocytic leukemia HL-60 and human acute Jurkat T cell leukemia cell lines were grown in 90% RPMI 1640 medium supplemented with 10% fetal bovine serum (FBS) at a temperature of 37 °C under a humidified 5% CO_2_ atmosphere. These cell lines were approved by short tandem repeat profiling, and the cells were passaged for no more than 2 months.

### 2.3. Cell Viability and Acridine Orange Staining Assay

Cells (2 × 10^6^/mL) were seeded into 60-mm Petri dishes and incubated at 37 °C. The cells were harvested by treatment with 5 µM rottlerin. Viable cells were counted using the trypan blue exclusion method. The cell suspension was mixed on a slide with an equal volume of acridine orange solution (10 mg/mL). Green fluorescence was detected between 500 and 525 nm with a fluorescence microscope (Zeiss, Oberkochen, Germany).

### 2.4. Transfection

The ODC-empty vector (pcDNA3-ODC-empty) and ODC-overexpressing vector (pcDNA3-ODC-WT) were transfected into parental HL-60 cells according to calcium phosphate-mediated transfection. Stably transfected cells were selected with the antibiotic G418 (400 μg/mL). After three weeks, the isolated G418-resistant clones were individually selected to analyze the protein expression of ODC in the cells.

### 2.5. DNA Fragmentation Assay

Cells were harvested and lysed overnight in digestion buffer (0.5% sarkosyl, 0.5 mg/mL proteinase K, 50 mM Tris-HCl, pH 8.0 and 10 mM ethylenediamine tetraacetic acid (EDTA) at 55 °C). Subsequently, cells were treated with 0.5 μg/mL RNase A for 2 h. Genomic DNA was extracted by phenol/chloroform/isoamyl alcohol extraction and analyzed by gel electrophoresis using 2% agarose.

### 2.6. Immunoblotting

To purify the total proteins, cells were harvested and lysed in cold lysis buffer (10% *v/v* glycerol, 1% *v/v* Triton X-100, 1 mM sodium orthovanadate, 1 mM ethylene glycol tetraacetic acid (EGTA), 10 mM NaF, 1 mM sodium pyrophosphate, 20 mM Tris, pH 7.9, 100 μM β-glycerophosphate, 137 mM NaCl, 5 mM EDTA, 1 mM phenylmethylsulfonyl fluoride (PMSF), 10 μg/mL aprotinin, and 10 μg/mL leupeptin), homogenized, and centrifuged, and then the supernatant was boiled in loading buffer with an aliquot corresponding to 50 μg of protein. The samples were then separated by SDS-PAGE and transferred to polyvinylidene difluoride (PVDF) membranes for immunoblotting using anti-ODC (sc-390366, Santa Cruz Biotechnology Inc., Dallas, TX, USA) or actin (mouse monoclonal, Santa Cruz Biotechnology Inc., Dallas, TX, USA) antibodies for 6 h, followed by a 1 h incubation with a horseradish peroxidase-labeled secondary antibody.

### 2.7. Expression and Purification of Human ODC

The human ODC gene was subcloned into the pQE30 vector, which carries an N-terminal His-tag sequence (Qiagen, Hilden, Germany). The protein expression and purification protocols have been reported in our previous papers [39]. Briefly, the expression vector with the ODC gene was transformed into the JM109 strain of Escherichia coli, and ODC protein overexpression was induced by 1 mM isopropyl-1-thio-b-D-galactoside (IPTG). The cells were grown at 25 °C, and the proteins were purified using Ni-NTA agarose (Sigma-Aldrich, St. Louis, MO, USA). Finally, the ODC protein was eluted with elution buffer (30 mM Tris-HCl, pH 7.6, 250–500 mM NaCl, 2 mM β-mercaptoethanol, and 250 mM imidazole), and the purified protein was dialyzed against dialysis buffer (50 mM Tris-HCl, pH 7.6, 0 or 500 mM NaCl, and 2 mM β-mercaptoethanol).

### 2.8. Enzyme Activity Assay and Inhibition Study of ODC

The ODC enzyme activity was measured at 37 °C using the CO_2_-L3K assay kit (DCL, Charlottetown, Canada) [39]. The enzyme assay couples the decarboxylation of ornithine to the carboxylation of phosphoenolpyruvate (PEP) to form oxaloacetate (OAA), which becomes malate following nicotinamide adenine dinucleotide (NADH) oxidation. The standard reaction mixture for the ODC spectrophotometric assay contained 30 mM Tris-HCl, pH 7.8, 10 mM ornithine, 0.2 mM pyridoxal phosphate (PLP), and 0.4 mL of CO_2_-L3K assay buffer containing 12.5 mM PEP, 0.4 units/mL microbial PEPC, 4.1 units/mL malate dehydrogenase (mammalian MDH), and 0.6 mM NADH analog in a final volume of 0.5 mL. An appropriate amount of ODC was then added to the assay mixture to initiate the reaction, and the decrease in absorbance at 405 nm was continuously traced with a Lambda 25 UV/VIS spectrophotometer (Perkin Elmer, Waltham, MA, USA). In the coupled reaction, 1 mol of CO_2_ was produced, and 1 mol of the NADH analog was oxidized under the assay conditions. One enzyme unit was defined as the amount of enzyme that catalyzes the production of 1 μmol of NAD per minute. An absorption coefficient of 2410 M^−1^ was used for the NADH analog in the calculations.

To determine the IC_50_ values of compounds that inhibited ODC, a dose-response curve was constructed with the following equation using SigmaPlot 10.0 software (Jandel, San Rafael, CA, USA).

To determine the *K*_i_ values of baicalein and 7,8-DHF, inhibition studies of ODC were conducted with different concentrations of baicalein (0, 0.5, 2, and 4 µM) or 7,8-DHF (0, 1, 2.4, and 8 µM) against a series of concentrations of ornithine. The total dataset was globally fitted using the following equation, which describes noncompetitive inhibition:

$$v=\frac{V_{\max}\times \left[S\right]}{\left(K_{m}+\left[S\right]\right)\times \;\left(1+\frac{\left[I\right]}{K_{i}}\right)}$$

where *v* is the observed initial velocity, *V*_max_ is the maximum rate of the reaction, *K*_m_ is the Michaelis constant for NAD^+^ or malate, and *K*_i_ is the inhibition constant for baicalein or 7,8-DHF.

### 2.9. Measurement of Cellular ODC Activity

The enzyme activity of ODC in cells was determined by measuring the increased level of putrescine after adding the substrate L-ornithine based on a luminescence-based coupled reaction assay [4,40]. The lysate protein (0.5–1 mg) was incubated with 1 mM L-ornithine and 0.5 mM pyridoxal-5′-phosphate (PLP) or only with 0.5 mM PLP, which was used as the blank representing the endogenous level of putrescine. The samples were incubated at 37 °C for 1 h, and the product putrescine was then detected by a PARADIGM Detection Platform microplate reader (Beckman Coulter, Atlanta, GA, USA) in luminescence detection mode. The luminescence of putrescine ranging from 0–40 pmol was determined as the standard, and the increased putrescine levels per hour represent the enzyme activity of ODC (pmol putrescine/mg lysate/h) in the cell.

### 2.10. In Silico Molecular Docking

The docking software PyRx version 0.97, together with AutoDock Vina, was used for all docking calculations [41]. The ligand structures, including baicalein (ZINC3871633), 7,8-DHF (ZINC57657) and myricetin (ZINC3874317), were obtained from the ZINC database (https://zinc.docking.org/). The receptor structure was obtained from the crystal structure of human ornithine decarboxylase from the RCSB Protein Databank (http://www.rcsb.org/) (PDB: 1D7K). Subsequent protein-ligand docking simulations were performed using PyRx. PyRx/AutoDock Vina is based on the Lamarckian genetic algorithm and empirical free energy scoring function. The receptor structure and ligands were prepared, docking was performed into a grid box space with the X, Y and Z axes, and the dimensions were adjusted to 49.43 Å × 38.97 Å × 40.61 Å. The exhaustiveness parameter was set to 20, and the number of modes was set to 9 in the PyRx platform. AutoDock Vina automatically samples different conformations of the ligands to best fit the predicted binding site. The docking results were analyzed based on the binding affinity (kcal/mol) of the protein-ligand complex. The more negative the binding affinity is, the better the orientation of the ligand in the binding site is. Docked complexes were visualized and analyzed using the PyMOL Molecular Graphics System (Ver. 2.2 Schrödinger, Portland, OR, USA), and the interactions between protein and ligand were analyzed using the LigPlot program [42].

### 2.11. Statistical Analysis

All data are expressed as the means ± SE. Statistical analysis was performed using the 95% confidence interval and appropriate post testing from Student’s t-test or one-way ANOVA at significance levels of *p* < 0.05 (*), *p* < 0.01 (**), and *p* < 0.001 (***). One-way ANOVA followed by Turkey’s multiple comparisons test or t-tests was performed using GraphPad Prism version 8.0.0 for Windows, GraphPad Software, San Diego, CA, USA, www.graphpad.com.

## 3. Results and Discussion

### 3.1. Flavone and Flavonol Derivatives Strongly Suppress Human ODC Enzymatic Activity

**Figure 1:**
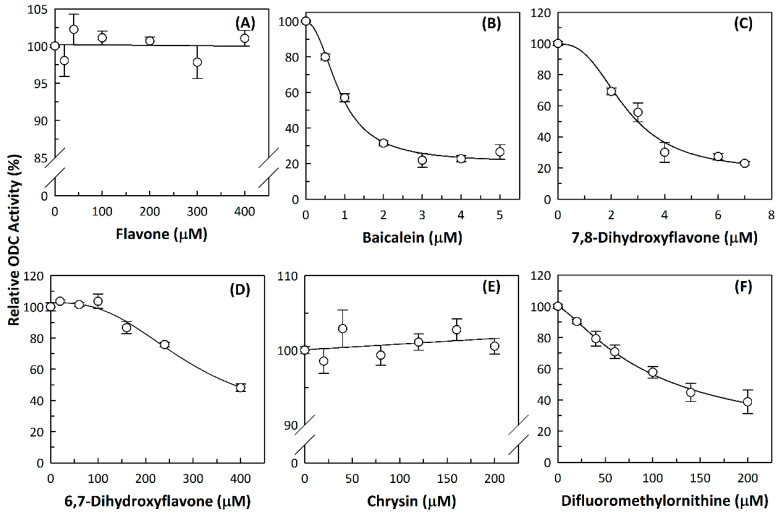
Dose-dependent inhibition of flavone derivatives toward the ornithine decarboxylase (ODC) enzyme. (**A**) Flavone; (**B**) baicalein; (**C**) 7,8-Dihydroxyflavone (7,8-DHF); (**D**) 6,7-Dihydroxyflavone (6,7-DHF); (**E**) chrysin; (**F**) difluoromethylornithine (DFMO), an irreversible inhibitor of ODC. Enzyme activity of ODC were measured with a series of concentrations of the respective flavone derivatives (*n* = 3).

**Figure 2:**
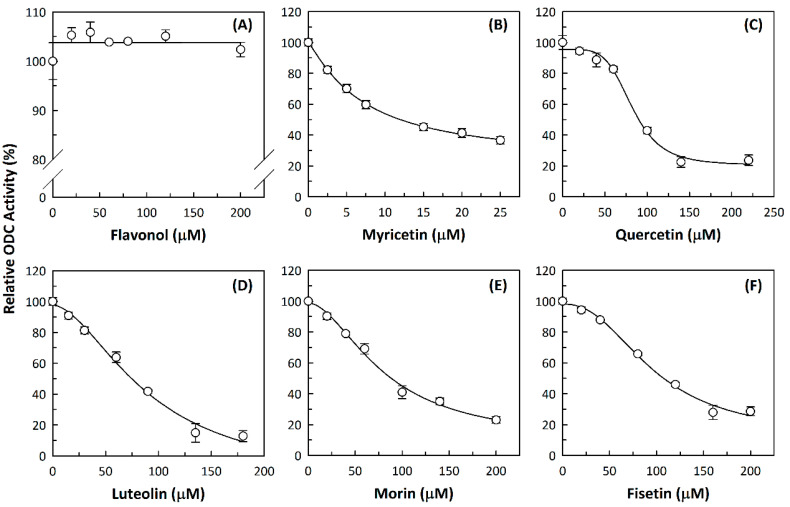
Dose-dependent inhibition of flavonol derivatives toward the ODC enzyme. (**A**) Flavonol; (**B**) myricetin; (**C**) quercetin; (**D**) luteolin; (**E**) morin; (**F**) fisetin. Enzyme activity of ODC were measured with a series of concentrations of the respective flavonol derivatives (*n* = 3).

We examined the inhibitory effects of a variety of flavone and flavonol derivatives on ODC enzymatic activity (Figure 1). Table 1 and Table 2 show the chemical structures of these derivatives along with their characteristics and IC_50_ values. Flavone and flavonol, without substitutions on the A or B rings, displayed little ODC suppression activity (Figure 1A and Figure 2A, respectively). Baicalein, a flavone with three consecutive hydroxyl groups at the 5th, 6th, and 7th positions of the A ring, exhibited significant ODC suppression activity with an IC_50_ of 0.88 µM (Figure 1B and Table 1). In addition, myricetin, a flavonol with three consecutive hydroxyl groups at the 3rd, 4th, and 5th positions of the B ring, exhibited substantial ODC suppression activity with an IC_50_ of 7.3 µM (Figure 2B and Table 2). The IC_50_ of DFMO determined in our system was approximately 100 µM (Figure 1F). As an enzyme inhibitor of ODC, baicalein is nearly 100-fold more potent than DFMO, and myricetin is nearly 10-fold more potent than DFMO.

### 3.2. Substitutions to the A or B Rings of the Flavone and Flavonol Derivatives Are Determining Factors for Human ODC Enzyme Suppression Activity

7,8-DHF, which has two consecutive hydroxyl groups at the 7th and 8th positions of the A ring, also exhibited unusual ODC suppression activity, with an IC_50_ of 2.54 µM (Figure 1C and Table 1). However, 6,7-DHF, which has two consecutive hydroxyl groups at the 6th and 7th positions of the A ring, displayed much weaker ODC-suppressing activity than 7,8-DHF; the IC_50_ of 6,7-DHF was 289 µM, approximately 100-fold greater than that of 7,8-DHF (Figure 1D and Table 1), indicating the significance of the positioning of the hydroxy groups. Although 7,8-dimethoxyflavone has two consecutive substituents (methyl groups) at the 7th and 8th positions of the A ring, this compound displayed little ODC suppression activity, indicating the crucial role of the hydroxyl groups (Figure S1A and Table 1).

Chrysin and pinocembrin are B ring stereoisomers that have two hydroxyl groups at the 5th and 7th positions of the A ring; however, they displayed little ODC suppression activity (Figure 1E and Figure S1B, respectively; Table 1). Therefore, it was not surprising that 6-hydroxyflavone and 7-hydroxyflavone, which each have a single hydroxyl group at the 6th or 7th position of the A ring, respectively, did not show any ODC suppression activity (Figure S1C,D; Table 1).

We also examined the ODC-inhibiting ability of other flavonols, including quercetin, luteolin, morin, rutin, kaempferol, apigenin, and biochanin (Figure 2C–E and Figure S2A–D, respectively). These flavonols have two hydroxyl groups at the 5th and 7th positions of the A ring, which have common features with myricetin but differ in the positioning and number of hydroxyl groups on the B ring (Table 2). Quercetin, luteolin and morin, which have two hydroxyl groups at the 4th and 5th positions, the 3rd and 4th positions or 2nd and 4th positions of the B ring, respectively, exhibited similar ODC suppression activity with IC_50_ values of approximately 80 to 90 µM, which were 10-fold greater than that of myricetin (Figure 2C–E; Table 2). Rutin, which has a similar structure to luteolin on the A and B rings but has many hydroxyl groups on the C ring, did not display apparent ODC-suppressing activity (Figure S2A and Table 2), suggesting that the ODC-inhibiting activity of flavone or flavonol derivatives is not due to the total number of hydroxyl groups present that may oxidize and modify the ODC protein. Fisetin is similar in structure to luteolin but lacks a single hydroxyl group on the 5th position of the A ring, and displayed an IC_50_ of 96 µM (Figure 2F and Table 2). Furthermore, kaempferol, apigenin, biochanin, daidzein, and naringenin, each of which have a single substitution on the B ring, did not show any ODC suppression activity (Figure S2B–F; Table 2).

Additionally, we tested the ODC-inhibiting ability of PLP analogs (4-deoxypyridoxine, 4-pyridoxic acid, and pyridoxamine) and other structurally related compounds (ethyl vanillin, 1,2-naphthoquinone, caffeine, sesamol, theobromine, and theophylline). However, none of these compounds were able to suppress ODC activity (Table S1), indicating that a chemical drug with a PLP-like structure is not sufficient to be an ODC inhibitor.

### 3.3. Baicalein and 7,8-DHF Demonstrate Noncompetitive Inhibition, and These Drugs May Bind to the Region near the Active Site Pocket at the Dimer Interface of ODC

**Figure 3:**
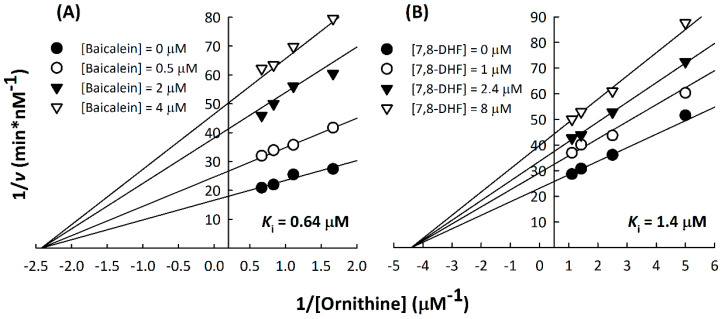
Non-competitive inhibition of human ODC by baicalein or 7,8-DHF. Double reciprocal plots for the inhibition of ODC with (**A**) baicalein and (**B**) 7,8-DHF with respect to the ODC substrate ornithine. Human ODC activity was measured using different concentrations of ornithine with various concentrations of baicalein (0, 0.5, 2, and 4 µM) or 7,8-DHF (0, 1, 2.4, and 8 µM).

The inhibition patterns of baicalein and 7,8-DHF with respect to the substrate L-ornithine were examined, and the inhibition constants values against ODC (*K*_i(ornithine)_) were determined. Kinetic analysis indicated that baicalein and 7,8-DHF displayed a noncompetitive inhibition pattern with respect to L-ornithine against ODC (Figure 3A,B). The *K*_i(ornithine)_ values of baicalein and 7,8-DHF against ODC were 0.64 ± 0.18 µM and 1.4 ± 0.1 µM, respectively, which were similar to their corresponding IC_50_ values (0.88 µM and 2.54 µM, respectively; Table 1).

**Figure 4:**
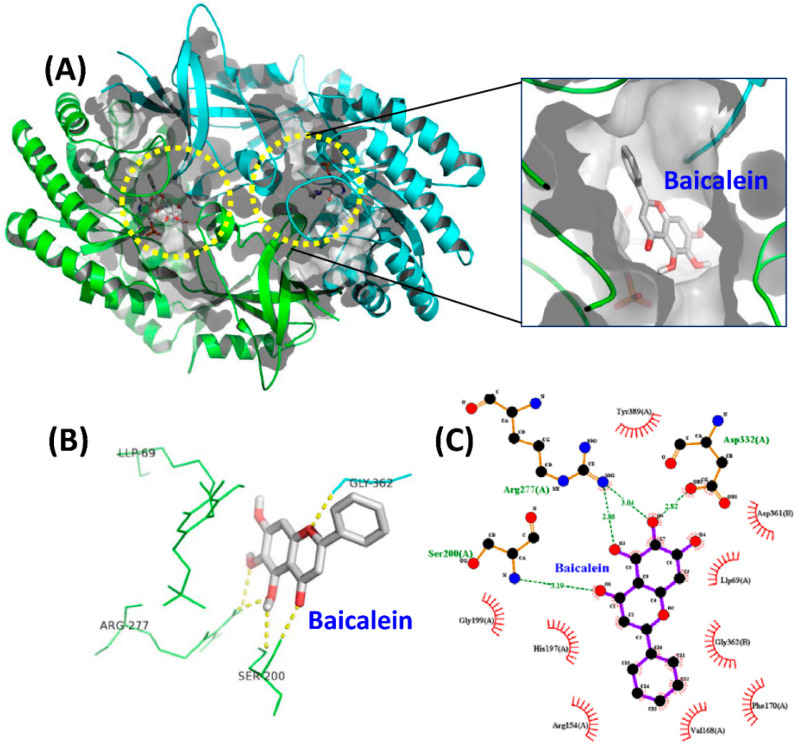
Molecular docking simulations of the ODC dimer with baicalein. (**A**) The best docking result of the ODC dimer with baicalein. The yellow circle indicates the binding pocket of baicalein. (**B**) Ligand interaction diagram of ODC with baicalein. (**C**) Ligand interactions between baicalein and ODC were drawn by the LigPlot program [41]. The green dotted line represents a hydrogen bond. Hydrophobic interaction contributions from nonligand residues are indicated by open spokes.

We performed in silico molecular docking of baicalein, 7,8-DHF and myricetin to the whole dimer of human ODC (PDB code: 1D7K) (Figure 4, Figures S3 and S4, respectively). Structural and functional studies revealed that residues Asp134, Thr157, Lys169, Val198, Ser200, Gly201, Arg277, Lys294, Tyr331, Asp332, Gly362, and Tyr389 are recognizable around the active site in the dimer interface and play significant roles in either dimer formation or enzymatic activity [39].

The docking results showed that baicalein was buried near the active site pocket in the dimer interface of the enzyme (yellow circles, Figure 4A). Baicalein was surrounded by amino acid residues from the A and B chains, which were hydrogen bonded to Ser200, Arg277, and Asp332 from the A chain and Gly362 from the B chain (Figure 4B,C, and Table 3). A negative binding energy of −8.3 kcal/mol was calculated for the binding of baicalein to ODC (Table 3), which coincided with the inhibition study since baicalein was determined to be a non-competitive ODC inhibitor with a *K*_i(ornithine)_ value of 0.64 µM; this result showed that baicalein is a high-affinity ODC inhibitor capable of strong binding to the enzyme. The simulation study showed that the hydroxyl moiety at C-6 on the A ring of baicalein concurrently acted as a hydrogen bond donor to Asp332 and an acceptor to Arg277; additionally, the oxygen on C-5 of the A ring accepted a hydrogen bond from Arg277. Since Arg277 and Asp332 are critical residues for ODC activity and dimerization, hydrogen bonding of baicalein with Arg277 and Asp332 may disturb the geometry of the active site in the dimer interface, thereby inhibiting ODC activity.

**Figure 5:**
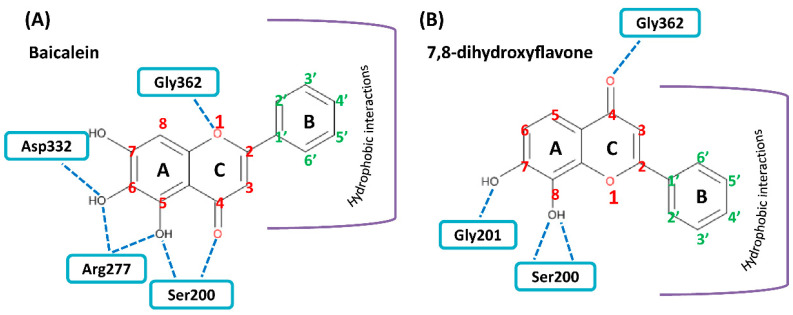
Schematic diagram for the chemical structure of baicalein and 7,8-DHF and the interacting residues of ODC. (**A**) Hydrogen bonding of the hydroxyl moieties at C-5 and C-6 on the A ring and the oxygen atom on the C ring of baicalein with Ser200, Arg277, Asp332, and Gly362 in the dimer interface of ODC. (**B**) Hydrogen bonding of the hydroxyl moieties at C-7 and C-8 on the A ring and the oxygen atom at C-4 on the C ring of 7,8-DHF with Ser200, Gly201, and Gly362 in the dimer interface of ODC.

7,8-DHF was also bound near the active site pocket of the dimer interface of the enzyme (yellow circles, Figure S3A). The molecular interactions were attributed to hydrogen bonding between the hydroxyl moiety at C-7 and C-8 on the A ring of 7,8-DHF with Gly201 and Ser200 of ODC, respectively (Figure S3B,C, and Table 3). A negative binding energy of −8.1 kcal/mol was observed for 7,8-DHF binding with the ODC dimer (Table 3), which acted as a high-affinity noncompetitive inhibitor with a *K*_i(ornithine)_ value of 1.4 µM. Interestingly, although the chemical structures of baicalein and 7,8-DHF and binding energies of these two compounds toward ODC are quite similar and both compounds displayed noncompetitive inhibition, the simulation study showed that the binding geometry of these two compounds in the enzyme were quite different (Figure 5). In 7,8-DHF, the oxygen atom at C-4 of the C ring forms a hydrogen bond with Gly362 (Figure 5B), but the oxygen atom at C-4 of the C ring of baicalein forms a hydrogen bond with Ser200 (Figure 5A). Additionally, Ser200 is hydrogen bonded to 7,8-DHF by the hydroxyl moiety at C-8 on the A ring (Figure 5B) and this same residue forms a hydrogen bond to baicalein through the hydroxyl moiety at C-5 on the A ring (Figure 5A). The basic structural elements that drive the potential inhibition of ODC may be as follows: the two consecutive hydroxyl moieties at C-5 and C-6 on the A ring and the oxygen atom within the C ring of baicalein, which are similar to the two consecutive hydroxyl moieties at C-7 and C-8 on the A ring and the oxygen atom at C-4 on the C ring of 7,8-DHF.

Myricetin also formed hydrogen bonds near the active site pocket in the dimer interface of the enzyme (yellow circles, Figure S4A) through its hydroxyl moieties on the A, B, and C rings that served as hydrogen bond donors or acceptors with the residues essential for ODC activity and dimerization (Figure S4B,C and Table 3). Among the three compounds, myricetin was shown to bind to ODC the tightest, with the lowest energy of −9.2 kcal/mol, but did not display the most effective inhibitory ability toward ODC, suggesting the specificity of the individual compounds toward the inhibition of ODC.

### 3.4. Baicalein and Myricetin Suppress Cell Growth and Induce Cellular Apoptosis of the Cell

**Figure 6:**
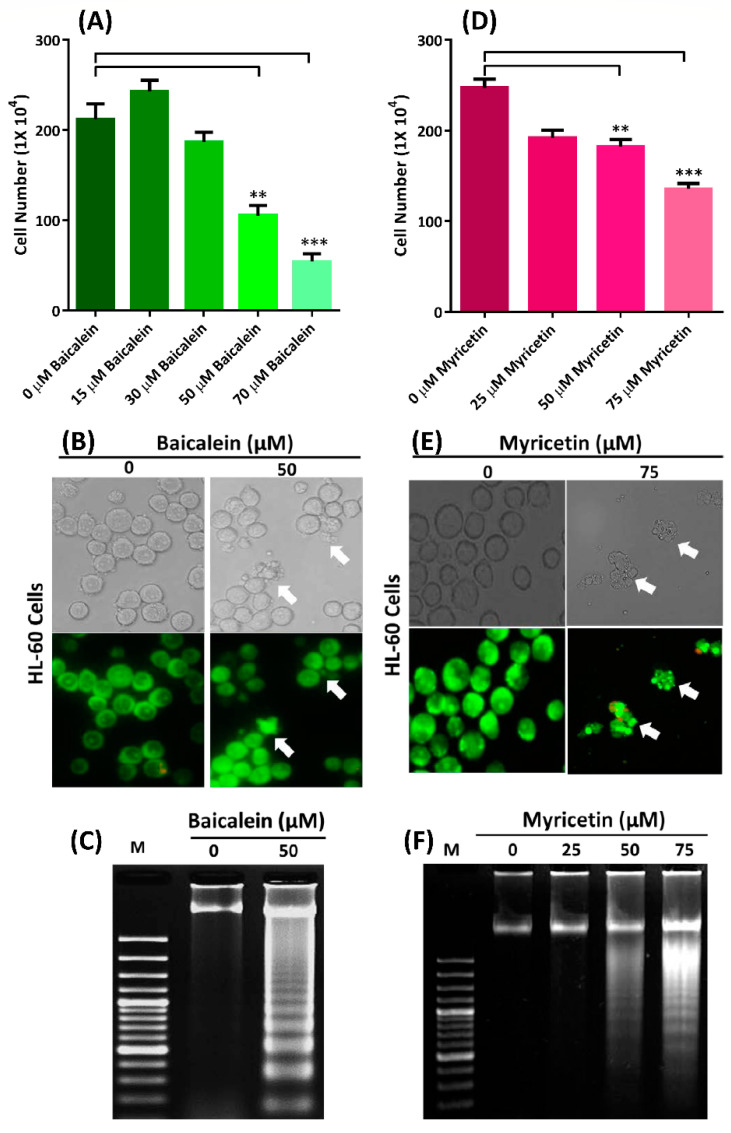
Baicalein and myricetin suppress cell growth and induce apoptosis of HL-60 cells. (**A**,**D**) Cell growth was suppressed in a dose-dependent manner with baicalein and myricetin, respectively. ** *p* < 0.01, *** *p* < 0.001 (*n* = 3). (**B**,**E**) Apoptotic bodies (indicated by arrows) in the cells were present after treatment with 50 µM of baicalein and 75 µM myricetin, respectively. (**C**,**F**) DNA fragmentation in the cells after treatment with 50 µM baicalein and 0-75 µM myricetin.

When HL-60 cells were treated with baicalein at concentrations of 0–70 μM, cell death was dose-dependent (Figure 6A). The IC_50_ value for baicalein in HL-60 cells after 24 h was approximately 50 μM (Figure 6A), and when these cells were treated with 50 μM baicalein, the cells exhibited apoptosis by displaying lobular apoptotic cell bodies (arrows in Figure 6B) and DNA fragmentation (Figure 6C). A similar result was observed in myricetin-treated HL-60 cells. The IC_50_ of myricetin in HL-60 cells after 24 h was approximately 75 μM (Figure 6D). Myricetin induced apoptosis of HL-60 cells at 24 h with a clearly visible apoptotic cell bodies (arrows in Figure 6E), and the DNA fragmentation of HL-60 cells occurred in a dose-dependent manner after HL-60 cells were treated with various concentrations of myricetin (Figure 6F). Additionally, both baicalein and myricetin suppressed the growth of Jurkat cells and induced cellular apoptosis with DNA fragmentation (Figure S5), indicating the cytotoxicity of these compounds toward leukemic cells.

### 3.5. Baicalein and Myricetin Suppress ODC-Evoked Cellular Anti-Apoptosis

**Figure 7:**
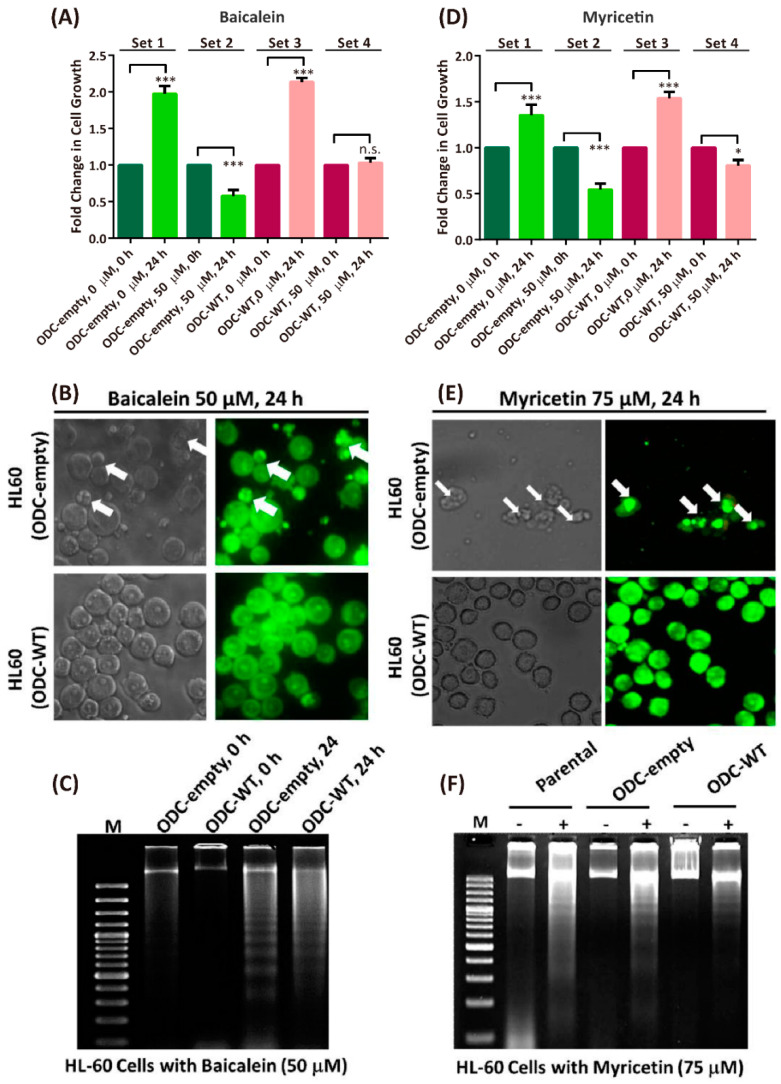
Baicalein and myricetin battled against ODC-evoked cellular anti-apoptosis. (**A**,**D**) Fold change in growth of HL-60 cells with ODC-empty vector (ODC-empty) and ODC-overexpressing vector (ODC-WT) in the absence or presence of 50 µM baicalein or 75 µM myricetin, respectively. * *p* < 0.01, *** *p* < 0.001 (*n* = 3). (**B**,**E**) Apoptotic bodies in the ODC-empty and ODC-WT HL-60 cells (indicated by arrows) were present after treatment with 50 µM baicalein and 75 µM myricetin, respectively. (**C**,**F**) DNA fragmentation in the ODC-empty and ODC-WT HL-60 cells (indicated by arrows) was present after treatment with 50 µM baicalein and 75 µM myricetin, respectively.

To determine whether baicalein- or myricetin-induced apoptosis was related to ODC expression, we introduced ODC cDNA into the pcDNA3 mammalian expression plasmid to produce the ODC-empty vector (ODC-empty) and ODC-overexpressing vector (ODC-WT) in parental HL-60 cells. Therefore, the protein expression and enzyme activity of ODC were expressed at a higher level in ODC-WT cells than in the parental HL-60 and ODC-empty cells (Figure S6). Without baicalein, both ODC-empty and ODC-WT HL-60 cells grew very fast, approximately 2- to 2.1-fold after 24 h (Set 1 and Set 2, respectively, Figure 7A). However, when ODC-empty or ODC-WT HL-60 cells were treated with 50 μM baicalein, the cells exhibited growth arrest within 24 h (Set 3 and Set 4, respectively, Figure 7A). The baicalein-induced growth arrest of the ODC-empty HL-60 cells coincided with the appearance of apoptotic bodies and DNA fragmentation (upper panels of Figure 7B,C). Furthermore, ODC-WT HL-60 cells exhibited a normal cell morphology and slight DNA fragmentation (lower panels of Figure 7B,C), indicating that overexpression of ODC could prevent baicalein-induced apoptosis.

Similar to baicalein, myricetin had cytotoxic effects toward ODC-empty or ODC-WT HL-60 cells. Myricetin-inhibited cell growth in ODC-empty HL-60 cells was more effective than in ODC-WT HL-60 cells (Set 3 and Set 4, respectively, Figure 7D). Myricetin treatment induced apoptotic bodies in ODC-empty cells but not in ODC-WT HL-60 cells (Figure 7E). In addition, myricetin-induced DNA fragmentation in parental and ODC-empty cells was more evident than that in ODC-WT HL-60 cells (Figure 7F). These data further supported that baicalein and myricetin are able to suppress ODC-induced anti-apoptotic effects in HL-60 cells.

### 3.6. Baicalein, 7,8-DHF, and Myricetin Can Be Used as Chemopreventive Agents by Targeting the ODC Enzyme

ODC is highly expressed in many cancers, promoting cell growth and tumorigenesis [43,44]. Elevated ODC activity in the cell leads to uncontrolled levels of cellular polyamines, and polyamines are critical for DNA folding and replication and cell proliferation [44,45]. Therefore, ODC has been considered a valid target for chemoprevention and cancer treatment. A well-known ODC irreversible inhibitor, DFMO, is an FDA-approved drug for cancer treatment. However, high doses of DFMO in humans will lead to different degrees of hearing loss. Our present study revealed several small molecule inhibitors of ODC from natural sources that are nontoxic and more potent than DFMO inhibitors of ODC activity and therefore could be good candidates as substitutes for DFMO to specifically inhibit ODC enzymatic activity.

## 4. Conclusions

Our present study indicated that the flavonoids baicalein, 7,8-DHF, and myricetin could effectively inhibit ODC activity, suppress cell growth and induce cellular apoptosis. Therefore, we suggest that these three compounds are potent chemopreventive and chemotherapeutic agents that work by targeting ODC. Besides three flavonoids we report in this paper, herbacetin, a natural flavonoid from flaxseed, has also demonstrated as an allosteric ODC inhibitor [31]. Another natural product allicin also demonstrates potent ODC-inhibitory effect, thus this natural product is also an ODC inhibitor potentially used as chemopreventive or chemotherapeutic agents [46]. Since ODC and polyamines are highly associated with the initiation of tumorigenesis, these nature products may be used as promising lead compounds for the development of pharmaceutical anticancer agents or as daily nutraceutical supplements to prevent cancer initiation and promotion. Further studies for the functional regulation of flavonoids baicalein, 7,8-DHF, and myricetin toward the homeostasis of cellular polyamines in various cancer cells that are overexpressed ODC will be discussed in the near future.

## Figures and Tables

**Table 1:** Flavonoids with A ring substitution(s) and with the the half maximal inhibitory concentration (IC_50_) to ornithine decarboxylase (ODC).

Compound	IC_50_	Chemical Structure	Characteristics
Flavone	No inhibition	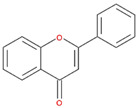	No A ring substitution
Baicalein (5,6,7-trihydroxyflavone)	0.88 μM	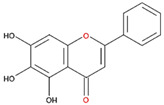	A ring substitution: 5,6,7-tri-OH
7,8-Dihydroxyflavone (7,8-DHF)	2.54 μM	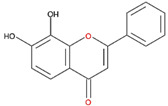	A ring substitution: 7,8-di-OH
6,7-Dihydroxyflavone (6,7-DHF)	289 μM	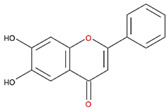	A ring substitution: 6,7-di-OH
Chrysin	No inhibition	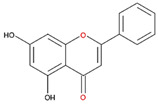	A ring substitution: 5,7-di-OH
7,8-Dimethoxyflavone	No inhibition	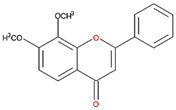	A ring substitution: 7,8-di-OCH_3_
Pinocembrin	No inhibition	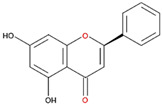	A ring substitution: 5,7-di-OH
6-Hydroxyflavone	No inhibition	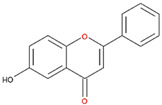	A ring substitution: 6-OH
7-Hydroxyflavone	No inhibition	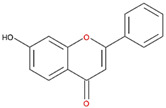	A ring substitution: 7-OH

**Table 2:** Flavonoids with substitutions on both the A and B rings and with the IC_50_ to ODC.

Compound	IC_50_	Chemical Structure	Characteristics
**Flavonol**	No inhibition	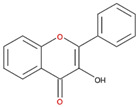	Flavonol No A or B ring substitutions
**Myricetin**	7.3 μM	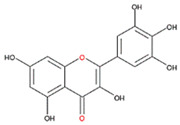	Flavonol A ring substitution: 5,7-di-OH B ring substitution: 3,4,5-tri-OH
**Quercetin**	82 μM	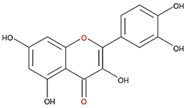	Flavonol A ring substitution: 5,7-di-OH B ring substitution: 4,5-di-OH
**Luteolin**	90 μM	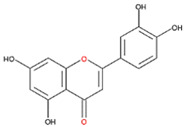	Flavone A ring substitution: 5,7-di-OH B ring substitution: 3,4-di-OH
**Morin**	82 μM	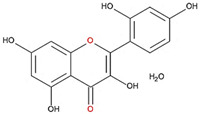	Flavonol A ring substitution: 5,7-di-OH B ring substitution: 2,4-di-OH
**Fisetin**	96 μM	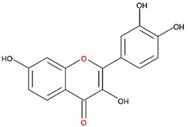	Flavonol A ring substitution: 7-OH B ring substitution: 3,4-di-OH
**Rutin**	>120 μM	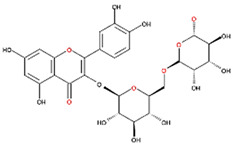	Flavonol derivative A ring substitution: 5,7-di-OH B ring substitution: 3,4-di-OH Many additional OH groups
**Kaempferol**	200 μM	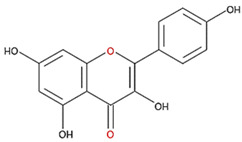	Flavonol A ring substitution: 5,7-di-OH B ring substitution: 4-OH
**Apigenin**	No inhibition	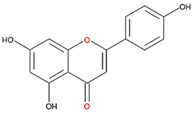	Flavone A ring substitution: 5,7-di-OH B ring substitution: 4-OH
**Biochanin**	No inhibition	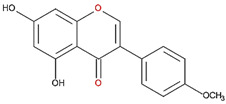	Isoflavone A ring substitution: 5,7-di-OH B ring substitution: 4-OCH_3_
**Daidzein**	No inhibition	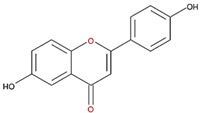	Flavone A ring substitution: 6-OH B ring substitution: 4-OH
**Naringenin**	No inhibition	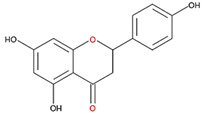	Flavone A ring substitution: 5,7-di-OH B ring substitution: 4-OH

**Table 3:** Molecular interactions between the ODC dimer and baicalein, 7,8-DHF, and myricetin.

Protein	Ligand	^1^ Binding Affinity (kcal/mol)	^2^ Number of Hydrogen Bonds	^2^ Hydrogen Bonding Residues	^2^ Interacting Hydrophobic Residues
ODC (1D7K)	Baicalein (ZINC3871633)	−8.3	4–6	Ser200(A), Arg277(A) Asp332(A), Gly362(B)	Arg154(A), Val168(A) Phe170(A), His197(A) Gly199(A), Tyr389(A) Asp361(B), Gly362(B)
	7,8-DHF (ZINC57657)	−8.1	3–4	Ser200(A), Gly201(A) Gly362(B)	Val198(A), Gly199(A) Tyr331(A), Asp332(A) Tyr389(A) Tyr323(B), Asp361(B)
	Myricetin (ZINC3874317)	−9.2	9–10	Cys360(A), Gly362(A) Arg154(B), Thr157(B) Val198(B), Arg277(B) Asp332(B), Tyr389(B)	Asp361(A) Phe170(B), His197(B)

^1^ Binding affinity of the docked complex was determined with PyRx/AutoDock Vina. ^2^ The numbers of hydrogen bonds, hydrogen bonding residues, and interacting hydrophobic residues of the docked complex were analyzed with the PyMOL and LigPlot programs and are visualized in Figure 4, Figures S3 and S4.

## Supplementary Materials

The following are available online at https://www.mdpi.com/2072-6643/12/12/3867/s1, Figure S1. Dose-dependent inhibition of flavone derivatives toward the ODC enzyme. Figure S2. Dose-dependent inhibition of flavonol derivatives toward the ODC enzyme. Figure S3. Molecular docking simulations of the ODC dimer with 7,8-DHF. Figure S4. Molecular docking simulations of the ODC dimer with myricetin. Figure S5. Baicalein and myricetin suppress cell growth and induce apoptosis in Jurkat cells. Figure S6. Protein levels and enzyme activity of ODC in ODC-overexpressing HL-60 cells. Table S1. PLP analogs and other compounds that cannot inhibit ODC activity
