# Supplementary material for: Baicalein, 7,8-Dihydroxyflavone and Myricetin as Potent Inhibitors of Human Ornithine Decarboxylase
Source: Nutrients. 2020 Dec 17;12(12):3867. doi: 10.3390/nu12123867 (PMC7765794; doi:10.3390/nu12123867)
Supplement: Supplementary file 1 [file nutrients-12-03867-s001.pdf]

Baicalein, 7,8-Dihydroxyflavone and Myricetin as Potent Inhibitors of Human Ornithine Decarboxylase

Yun-Chin Liu, Yi-Liang Liu, Ju-Yi Hsieh, Chang-Hsu Wang, Chi-Li Lin, Guang-Yaw Liu, and Hui-Chih Hung

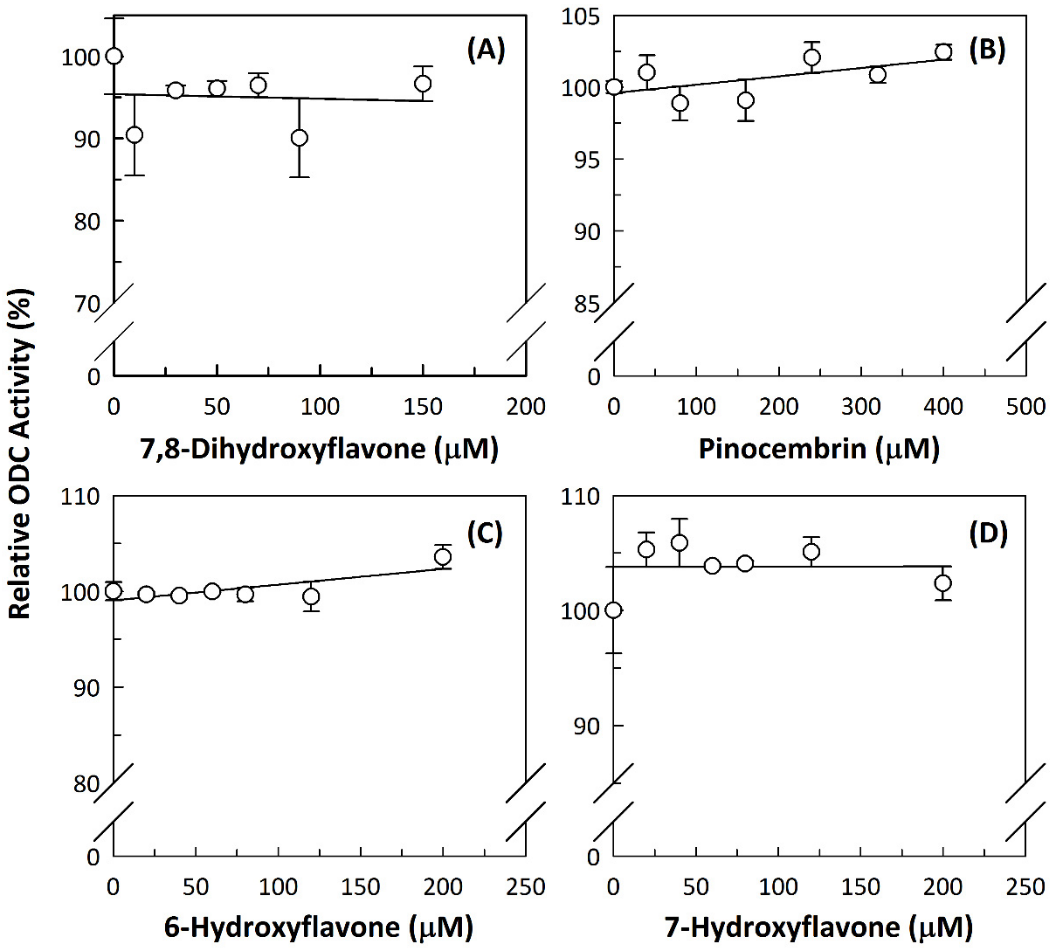

Figure S1. Dose-dependent inhibition of flavone derivatives toward the ODC enzyme. (A) 7,8-dimethoxyflavone; **(B)** pinocembrin; **(C)** 6-hydroxyflavone**; (D)** 7-hydroxyflavone. Enzyme activity of ODC were measured with a series of concentrations of the respective flavone derivatives (n =3).

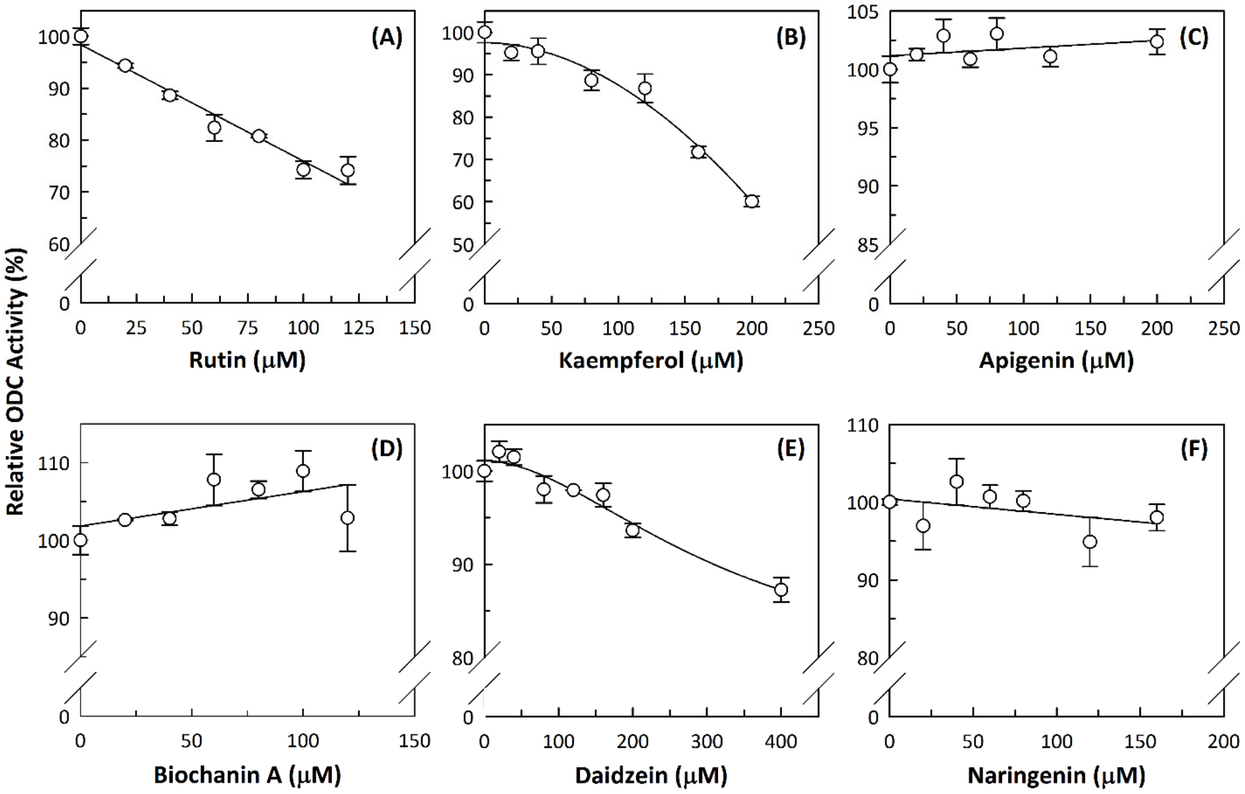

Figure S2. Dose-dependent inhibition of flavonol derivatives toward the ODC enzyme. (A) Rutin; **(B)** kaempferol; **(C)** apigenin **(D)** biochanin; **(E)** daidzein; **(F)** naringenin. Enzyme activity of ODC were measured with a series of concentrations of the respective flavonol derivatives (n =3).

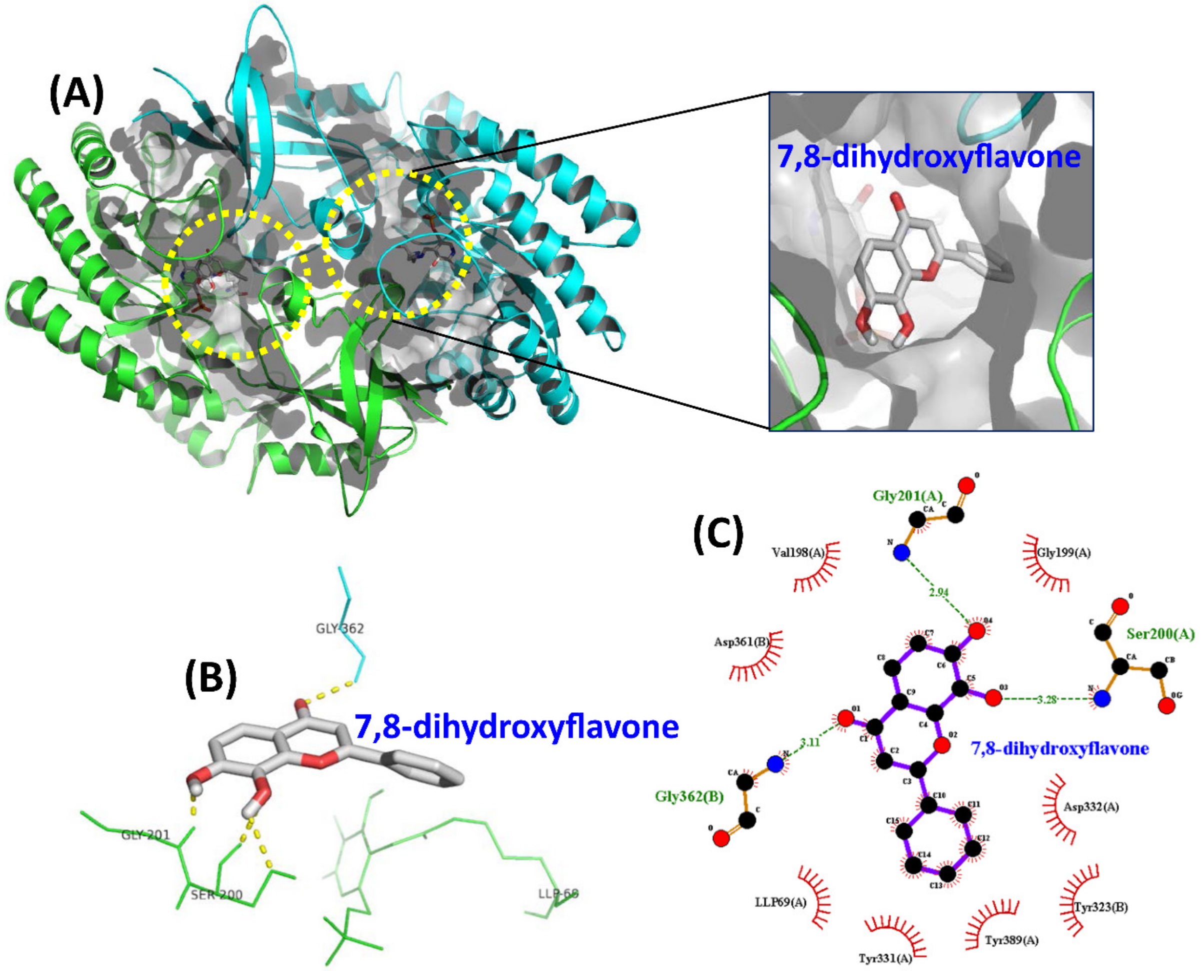

Figure S3. Molecular docking simulations of the ODC dimer with 7,8-DHF. (A) The best docking result of the ODC dimer with 7,8-DHF. The yellow circle indicates the binding pocket of 7,8-DHF. **(B)** Ligand interaction diagram of ODC with 7,8-DHF. **(C)** Ligand interactions between 7,8-DHF and ODC were drawn with the LigPlot program [41]. The green dotted line represents a hydrogen bond. Contributing hydrophobic interactions from nonligand residues are indicated by open spokes.

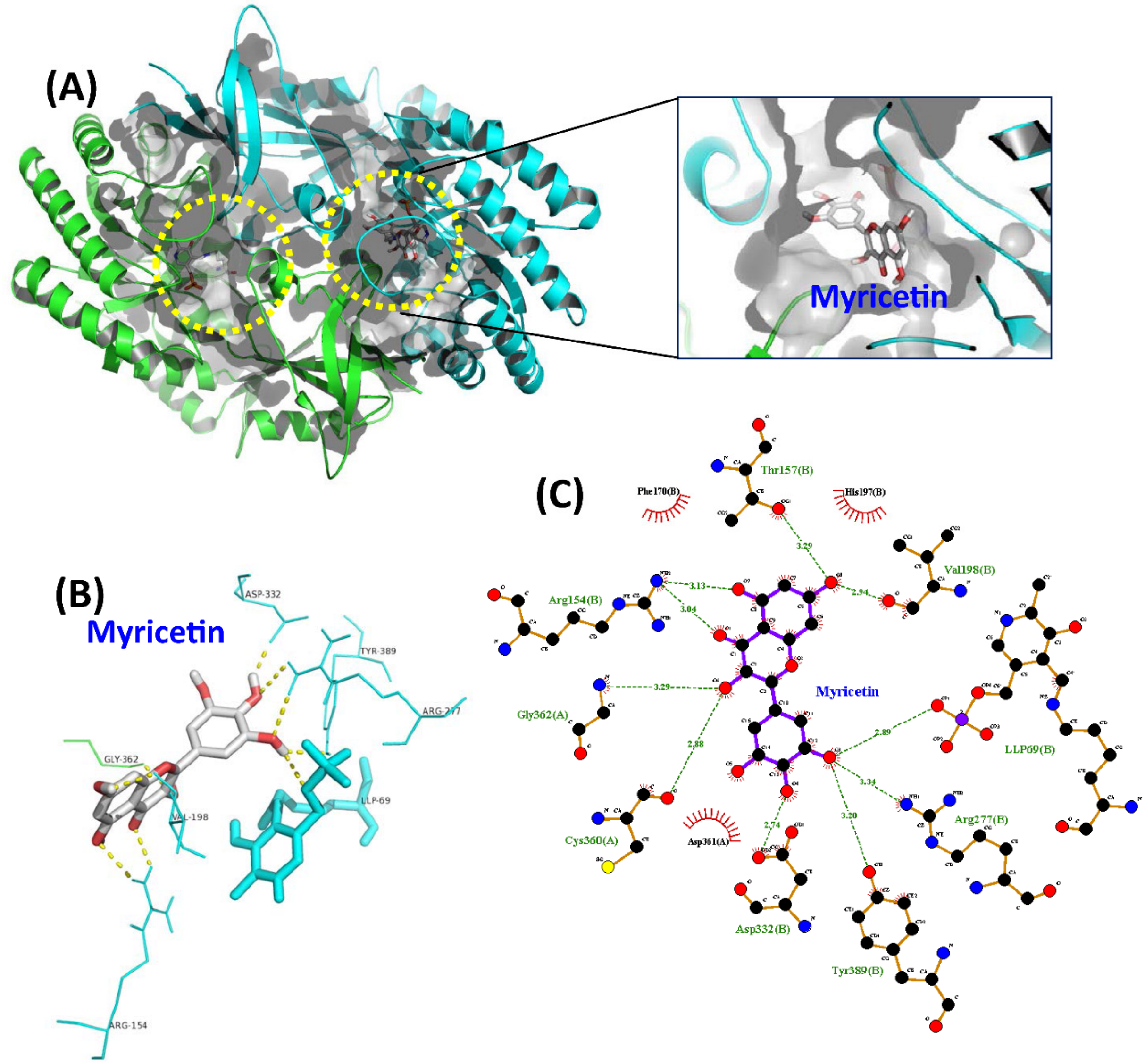

Figure S4. Molecular docking simulations of the ODC dimer with myricetin. (A) The best docking result of the ODC dimer with myricetin. The yellow circle indicates the binding pocket of myricetin. **(B)** Ligand interaction diagram of ODC with myricetin. **(C)** Ligand interactions between myricetin and ODC were drawn by the LigPlot program [41]. The green dotted line represents a hydrogen bond. Contributing hydrophobic interactions from nonligand residues are indicated by open spokes.

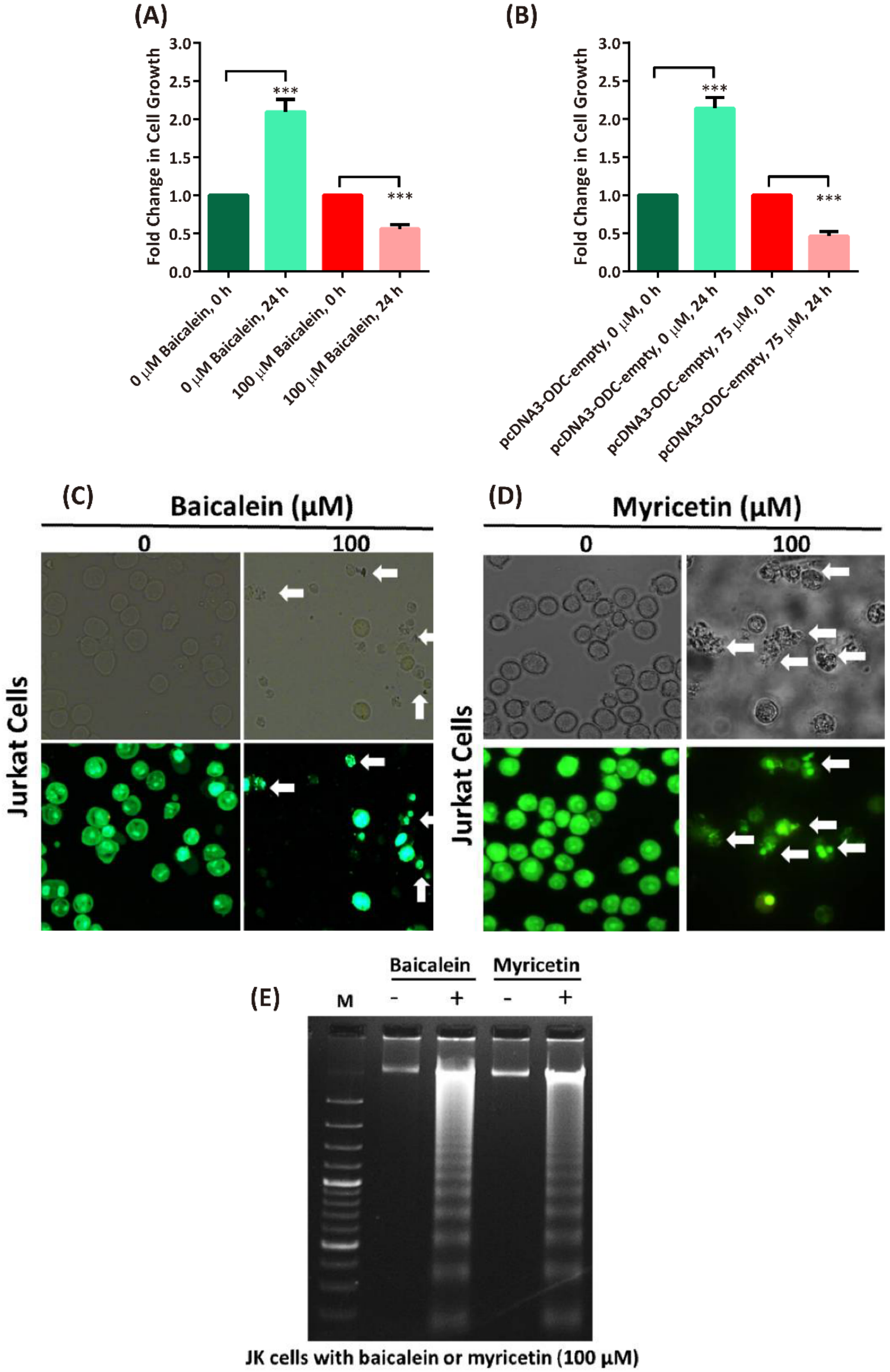

Figure S5. Baicalein and myricetin suppress cell growth and induce apoptosis in Jurkat cells. (A) and **(B)** Fold change in growth of Jurkat cells with in the absence or presence of 100 μM baicalein or 75 μM myricetin, respectively. **$p < 0.01$, ***$p < 0.001$ (n = 3). **(C)** and **(D)** Apoptotic bodies (indicated by arrows) in the cells were present after treatment with 100 μM baicalein or myricetin, respectively. **(E)** DNA fragmentation in the cells after treatment with 100 μM baicalein or myricetin.

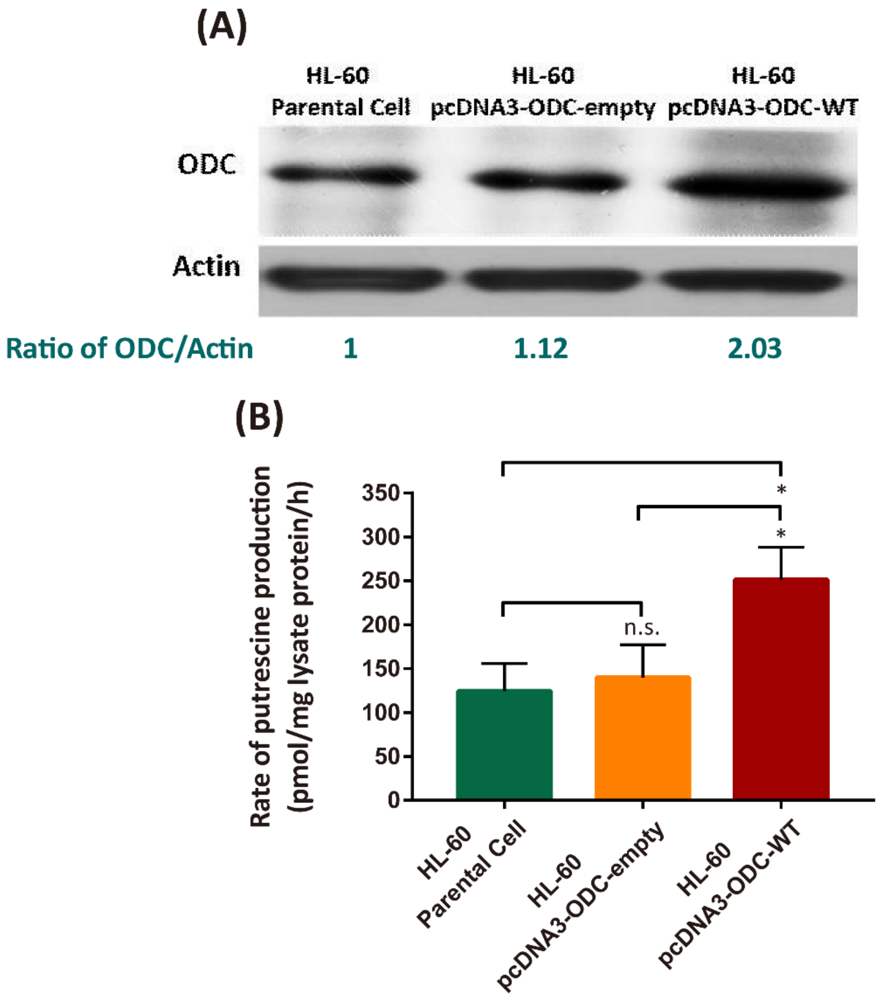

Figure S6. Protein levels and enzyme activity of ODC in ODC-overexpressing HL-60 cells. (A) The ODC protein was immunoblotted to indicate the level of ODC protein in the parental, pcDNA3-ODC-empty and pcDNA3-ODC-WT HL-60 cells (n = 2). Actin was used as the internal control. The amount of ODC was relative to its actin, and the relative ratio of ODC was shown. **(B)** Cellular ODC enzyme activity was estimated by measuring putrescine production within 1 h. *$p < 0.05$ (n = 3).

Table S1. PLP analogs and other compounds that cannot inhibit ODC activity

Compound/Chemical Structure		
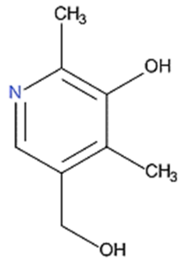 **4-Deoxypyridoxine**	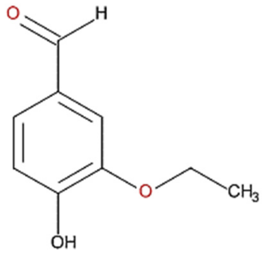 **Ethyl vanillin**	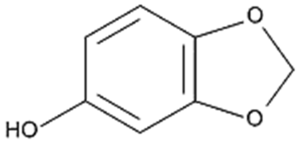 **Sesamol**
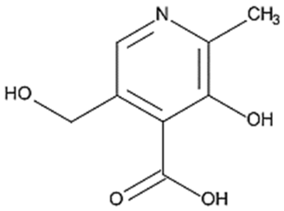 **4-Pyridoxic acid**	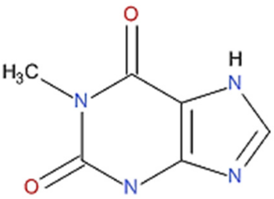 **1,2-Naphthoquinone**	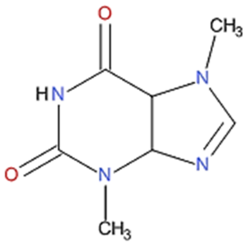 **Theobromine**
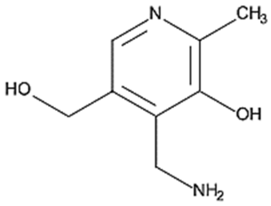 **Pyridoxamine**	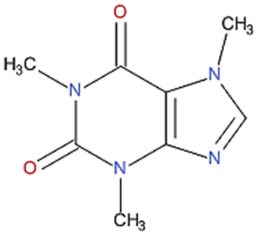 **Caffeine**	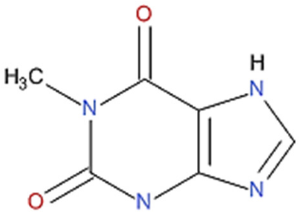 **Theophylline**
